# Health Potential of *Aloe vera* against Oxidative Stress Induced Corneal Damage: An “In Vitro” Study

**DOI:** 10.3390/antiox10020318

**Published:** 2021-02-20

**Authors:** Ida Ceravolo, Federica Mannino, Natasha Irrera, Francesco Squadrito, Domenica Altavilla, Giorgia Ceravolo, Giovanni Pallio, Letteria Minutoli

**Affiliations:** 1 Department of Clinical and Experimental Medicine, University of Messina, Via C. Valeria, 98125 Messina, Italy; ceravoloida@gmail.com (I.C.); fmannino@unime.it (F.M.); nirrera@unime.it (N.I.); fsquadrito@unime.it (F.S.); lminutoli@unime.it (L.M.); 2 Department of Biomedical, Dental, Morphological and Functional Imaging Sciences, University of Messina, Via C. Valeria, 98125 Messina, Italy; daltavilla@unime.it; 3 Department of Human Pathology and Evolutive Age “Gaetano Barresi”, University of Messina, Via C. Valeria, 98125 Messina, Italy; giorgiaceravolo@gmail.com

**Keywords:** ROS, oxidative stress, inflammation, apoptosis, corneal epithelial cells, *Aloe vera*, antioxidant

## Abstract

Fuchs endothelial corneal dystrophy (FECD) is characterized by the gradual deterioration of corneal endothelial cells (CECs) and is the most common cause of corneal transplantation worldwide. CECs apoptosis caused by oxidative stress plays a pivotal role in the pathogenesis of FECD. Antioxidant compounds have been of considerable significance as a candidate treatment in the management of corneal diseases. Based on these findings, the objective of this study was to evaluate the effects of an aloe extract with antioxidant properties, in an “in vitro” model of FECD. Human corneal epithelial (HCE) cells were preincubated with aloe extract 100 μg/mL, two hours before hydrogen peroxide (H_2_O_2_) stimulus. H_2_O_2_ challenge significantly reduced the cell viability, increased the generation of Reactive Oxygen Species (ROS) and malondialdehyde levels. Moreover, m-RNA expression and activity of Nrf-2, Catalase and Superoxide dismutase (SOD) were reduced together with an enhanced expression of IL-1β, tumor necrosis factor-α (TNF-α), IL-6, and cyclooxygenase 2 (COX-2). Furthermore, Bcl-2, Caspase-3 and Caspase-8 expression were down-regulated while Bax was up-regulated by H_2_O_2_ stimulus. Aloe extract blunted the oxidative stress-induced inflammatory cascade triggered by H_2_O_2_ and modulated apoptosis. Aloe extract defends HCE cells from H_2_O_2_-induced injury possibly due its antioxidant and anti-inflammatory activity, indicating that eye drops containing aloe extract may be used as an adjunctive treatment for FECD.

## 1. Introduction

Fuchs endothelial corneal dystrophy (FECD) is a bilateral hereditary and slowly progressive disease which affects the endothelial layer of the cornea of both eyes. The prevalence of FECD is approximately four percent of the adult population. Early manifestation can be observed in patient from 30 to 40 years old but Fuchs usually become symptomatic over 40 years of age or later. Women are more commonly affected than men are. In addition, family history increases the chance of developing the disease. Symptoms are usually represented by blurred and cloudy vision, especially in the morning that improves during the day, photophobia and feeling of foreign body inside the eye. Fuchs dystrophy can be classified in four stages, which vary from early signs of guttae formation to end-stage subepithelial scarring. Diagnosis is confirmed through ophthalmological examination, corneal pachymetry ad in-vivo confocal. It is characterized by the gradual deterioration of corneal endothelial cells (CECs) developing a characteristic feature (cornea guttate) and is the most common cause of corneal transplantation worldwide [1,2]. The deterioration of corneal endothelial cells and the consequent guttae formation usually start in the centre of the cornea and slowly involves the periphery as well. The longer the guttae spreads, the more CECs are destroyed and the density of endothelial cells decreases, as these two parameters are inversely proportional.

In this stage, the changes in Fuchs endothelial cells include a modification of the cell size called “polymegethism” and a modification of the cell outline called “pleomorphism”. The corneal endothelium (CE) is a monolayer formed by the CECs situated on the corneal surface that has different functions including: maintaining corneal deturgescence thanks to his barrier function and regulating corneal hydration, nutrition and transparency. As the CE does not divide in vivo, loss of endothelial cells seen in FECD is permanent. CECs apoptosis caused by oxidative stress may play a pivotal role in the pathogenesis of FECD [3,4]. Previous studies have already demonstrated a higher level of reactive oxygen species (ROS) in the cornea of FECD patients when compared with the healthy one [5]. The imbalance between oxidant and antioxidant factors in CECs is responsible for the development of endothelial oxidative DNA damage. It is caused by a decreased expression of Nrf2 transcription factor and its antioxidant targets like superoxide dismutases, glutathione S-transferases and peroxiredoxin, which are involved in the scavenging of ROS. The aberrant Nrf2 expression influences the antioxidant system in FECD corneal endothelium and induces free radicals and other reactive species to accumulate, leading to an alteration of tissue homeostasis and activating the p53-dependent apoptotic pathway [6,7].

Reactive oxygen species could be produced after photochemical reactions caused by the exposure to UV light or ionizing radiation. During physiological conditions, there is a cellular equilibrium between ROS production and degradation and low levels of ROS can be found [8]. The imbalance between ROS production and the antioxidant scavenging systems (AOX) causes Oxidative stress (OS). OS is related to several disorders, including Parkinson’s and Alzheimer’s diseases, cancer, atherosclerosis, diabetes and rheumatoid arthritis [9,10,11]. Furthermore, OS is also responsible for different ocular pathologies, such as ocular surface disorders, different syndromes of the eye anterior and posterior segment and retinal diseases. The cornea is a transparent and avascular tissue in the anterior segment of the eye and is one of the most densely innervated tissues in the body with refractive and barrier functions [12]. Due to its external localization, it is directly exposed to different factors, such as air pollution, cigarette smoke and UV radiations, which can induce oxidative damage, and different ocular pathologies such as FECD [13].

Corneal tissue has developed physiological antioxidant systems, which contain free radical scavengers, including superoxide dismutase, glutathione peroxidase and catalase [14]. The imbalance between prooxidant and antioxidant is primarily attributed to the down-regulation of the antioxidant enzymes principally: Lactate dehydrogenase, catalase and glutathione peroxidase. This imbalance leads to structural and functional changes in the corneal tissue. As a consequence of the increased oxidative stress there is a reduction of the number of corneal fibroblasts and corneal endothelial cells in corneas, due to the triggering of the apoptotic process [15,16,17]. Both elevated ROS levels and oxidative stress play a key role in the development of many corneal diseases, including Fuchs endothelial corneal dystrophy, keratoconus, granular corneal dystrophy type 2 and bullous keratopathy [18,19].

Previous papers have investigated the possibility of using phytotherapic treatments as new strategies for ROS related diseases [20,21]. Health plants are currently used in a number of consumer products due to their medicinal or aesthetic properties. Among them, the *Aloe vera* extracts are known for their curative properties, and are used both internally and externally on humans in the form of alternative medications, as well as in the home for first aid. *Aloe vera* has demonstrated wound-healing properties and showed immunomodulatory, antioxidant and anti-inflammatory effects [22]. Indeed, different studies showed that *Aloe vera* treatment induced inhibition of the inflammatory reactions, reducing the expression of proinflammatory cytokines, like interleukine-6 (IL-6), IL-1β and tumor necrosis factor-α (TNF-α) [23]. Moreover, *Aloe vera* extracts revealed a modulating effect on the inflammatory cytokine network and antioxidant function including ROS scavenging in eye tissue [24]. Furthermore, previous work established that the use of Nrf2 agonists with antioxidant activity caused cytoprotective effects, a significant decrease in ROS production and ameliorated oxidative stress-levels in FECD corneas, confirming the idea that the implementation of antioxidant system may play a role in the treatment of corneal ROS related diseases [25].

Finally, Jurkunas et al. demonstrated that immortalized normal corneal endothelial cells stimulated with H_2_O_2_ may display morphological changes and cellular apoptosis similar to those observed in FECD [5]. For all these reasons, the aim of the present study is to investigate the effects of an *Aloe vera* extract in an “in vitro” model of FECD induced by H_2_O_2_ stimulation in human corneal cells.

## 2. Materials and Methods

### 2.1. Cell Culture

Epithelial Adenovirus 12-SV40 hybrid transformed HCE cells (ATCC^®^ CRL-11135^™^) were obtained from LGC Standards S.r.l Milan, Italy. Cells were cultured in a medium prepared with DMEM-F12 basic media supplemented with 10% heat-inactivated FBS, recombinant human epidermal growth factor (5 ng/mL), 5 μg/mL insulin plus 1% antibiotics (penicillin/streptomycin), and incubated at 37 °C with 5% of CO_2_. The culture’s medium was replaced with a time interval of 2 days and the cells were re-plated.

### 2.2. Treatments of Cells

HCE cells were put in culture using six well plates and a density of 3 × 10^5^ cells/well. To establish an oxidative stress model 500 μM H_2_O_2_ (Sigma Aldrich, Milan, Italy) was added to the culture medium for 24 h. HCE cells were preincubated with aloe extract 100 μg/mL 2 h before H_2_O_2_ stimulus. HCE cells treated with 1% DMSO were used as control. The oxidative stress model, the doses and the incubation time of *Aloe vera* extract were chosen according to previously published papers [5,24,26,27].

### 2.3. MTT Assay

Cell viability was assessed using the MTT assay. HCE cells were treated with H_2_O_2_ (500 μM), H_2_O_2_ + aloe extract (100 μg/mL), when reaching confluence. In particular, CTRL, H_2_O_2_, H_2_O_2_ + aloe extract, were examined in a 96-well plate with a density of 8 × 10^4^ cells/well for 24 h in order to evaluate the cytotoxic effect as previously described [28,29,30].

### 2.4. Ros Measurament

To evaluate the effect of aloe extract on OS, the production of Total Reactive Oxygen Species (ROS) in HCE cells was measured using an assay kit (Thermo Fisher, Carlsbad, CA, USA). Briefly, after the treatments the HCE cells were cleaned with PBS once, and then were incubated at 37 °C for 30 min with 2′,7′-dichlorofluorescein diacetate. DCFH-DA fluorescence distribution of 1 × 10^4^ cells was detected using a flow cytometry at two different wavelength 488 nm for excitation and 525 nm for emission [13].

### 2.5. Malondialdehyde Assay

The effects of aloe extract as antioxidants against lipid peroxidation in HCE cells were examined using as marker the levels of malondialdehyde (MDA) as previously described in details [31,32,33].

### 2.6. Real Time Quantitative PCR Amplification (RT-qPCR)

The m-RNA expression of SOD2, Catalase, Nrf2, Il-1β, TNF-α IL-6, COX-2, Bcl-2, Bax, Caspase-3 and Caspase-8 was evaluated as previously described [34,35,36]. Primers used to identify both targets and reference genes are catalogued in Table 1.

### 2.7. Measurements of Cytokines

Nrf2, IL-1β, TNF-α, IL-6, PGE2 and Caspase-8 levels were measured in the cell culture supernatants, Bax, Bcl-2 and Caspase-3 levels were evaluated in the cell culture extracts using an Enzyme-Linked Immunosorbent Assay (ELISA) kits (Abcam, Cambridge, UK or Thermo Fisher, Waltham, MA, USA), in agreement with the instructions reported by the manufacturer [37,38,39,40].

### 2.8. Catalase and SOD Activity Measurement

The Catalase and SOD activity were evaluated in agreement with the manufacturer’s protocol of commercial kits (Thermo Fisher, Waltham, MA, USA). All the samples were measured in duplicate and the results were interpolated with the Catalase or SOD standard curve and the results were expresses in units/mL.

### 2.9. Collection of Plant Samples and Extract Preparation

*Aloe vera* plants (Aloe barbadensis Miller) were subjected to vegetative propagation as previously described [41]. Briefly, young shoots were cut from the mother plant and subsequently were planted in 0.5 kg plastic pots with incorporated commercial ground. Plants received tap water once every two weeks and grown under ambient irradiance of (400–1400 μmol m^−2^ s^−1^) and a temperature of (25 ± 1 °C). After 12 months of growth, the earliest fully developed leaves were used for the extract preparation. Fresh leaves were cleaned with distillated water and cut into fragments of around 20 g each. The extraction was performed by grinding a sample of 20 g with 100 mL of 100% methanol using a high performance grinder, followed by agitation for 4 h at 4 °C. After, the extract was evaporated in a bath at 60 °C and then lyophilized for 24 h The obtained powder was weighed and stored until the use.

### 2.10. Phytochemical Analysis of Aloe vera Extract

In *Aloe vera* plants, several phytochemical constituents are present such as: anthraquinones, fatty acids, alkaloids, carbohydrates, enzymes, vitamins, mineral and other miscellaneous compounds [20]. A phytochemical screening was performed the *Aloe vera* methanolic extract was subjected to for the presence or absence of various phytochemical according to standard protocols [42,43].

### 2.11. Statistical Analysis

Data are shown as the mean ± SD and the values reported are the result of at least five experiments performed in duplicate (two wells for each treatment). To ensure reproducibility, all assays were replicated three times. The various groups were compared and evaluated using one-way ANOVA with Tukey post-test for comparison between the different groups. A *p* value < 0.05 was considered significant. Graphs were prepared using GraphPad Prism (version 8.0 for macOS, San Diego, CA, USA).

## 3. Results

### 3.1. Phytochemical Screening of Aloe vera Extract

A wide range of various phytochemicals; alkaloids, flavonoids, glycosides, phenolic compounds, tannins and saponin, steroids and terpenoids, glycosides were tested with their appropriate protocols and reagents. The *Aloe vera* extract showed presence of most of the phytochemicals tested, the characterization is depicted in Table 2.

### 3.2. Effects of Aloe Extract on Cell Vitality and Oxidative Stress

**Figure 1:**
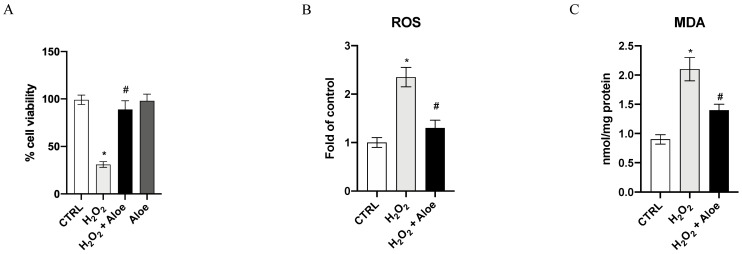
Effects of pre-treatment with aloe extract on cell viability (**A**), ROS production (**B**), MDA generation (**C**). Values are expressed as the means ± SD. * *p* < 0.0001 vs. CTRL; # *p* < 0.0001 vs. H_2_O_2_.

The viability of HCE cells was drastically reduced after exposure to 500 μM H_2_O_2_ for 24 h, as compared with the control group (*p* < 0.0001 versus control group; Figure 1A). Pre-treatment for 2h with aloe extract at 100 μg/mL significantly increased the cell viability of HCE cells incubated with H_2_O_2_ (*p* < 0.0001 vs. H_2_O_2_ group; Figure 1A). Furthermore, the incubation of aloe extract did not affect cell viability, thereby showing that this natural extract does not have a cytotoxic effect. To examine the ROS level in this oxidative stress setting and the effects of aloe extract, we measured the ROS production. An exposure to H_2_O_2_ at a concentration of 500 μM for 24 h resulted in high levels of ROS compared to the control group (*p* < 0.0001 vs. control group; Figure 1B). Whereas, aloe extract at a concentration of 100 μg/mL significantly suppressed the production of ROS (*p* < 0.0001 vs. H_2_O_2_ group; Figure 1B), indicating that aloe extract suppressed ROS production under the oxidative stress induced by H_2_O_2_. An important aspect of damage caused by ROS, especially by H_2_O_2_, is the oxidation of lipids, such as MDA. We observed a significant increase in MDA generation by H_2_O_2_ stimulus (*p* < 0.0001 vs. control group; Figure 1C). Moreover, aloe extract at 100 μg/mL significantly inhibited MDA levels compared to the H_2_O_2_ group (*p* < 0.0001 vs. H_2_O_2_ group; Figure 1C).

### 3.3. Effects of Aloe Extract on mRNA Expression and Activity of Antioxidant Markers

**Figure 2:**
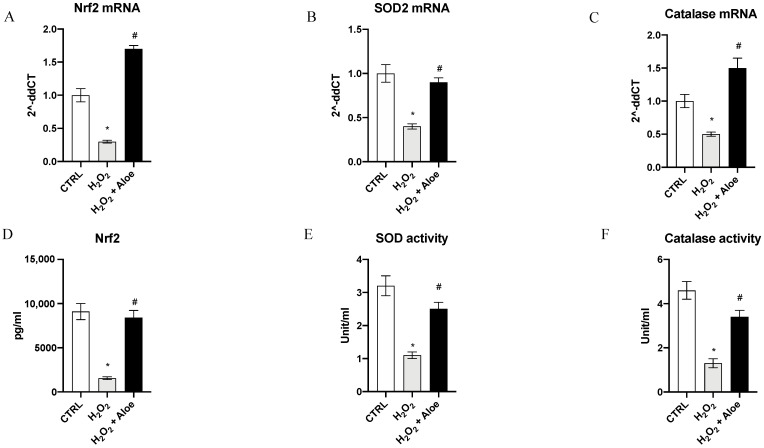
Effects of pre-treatment with aloe extract on Nrf2 (**A**), SOD2 (**B**), Catalase (**C**) mRNA expression. Effects of pre-treatment with aloe extract on Nrf2 levels (**D**), SOD activity (**E**), Catalase activity (**F**). Values are expressed as the means ± SD. * *p* < 0.0001 vs. CTRL; # *p* < 0.0001 vs. H_2_O_2_.

To analyze the effects of aloe extract during oxidative stress, we measured the gene expression and mature protein levels of Nrf2, chief regulator of the antioxidant system, and the enzyme activities and gene expression of SOD2 (one of the major antioxidant defense systems against free radicals) and catalase (one of the most important antioxidant enzymes) involved in antioxidant defenses. Exposure of HCE cells to H_2_O_2_ for 24 h significantly decreased mRNA expression of Nrf2, SOD2 and Catalase compared to the control group (*p* < 0.0001 vs. control group; Figure 2). While, 2h of pre-treatment with aloe extract significantly upregulated the gene expression of Nrf2, SOD2 and Catalase when compared to cell cultures challenged with H_2_O_2_ alone (*p* < 0.0001 vs. H_2_O_2_ group; Figure 2). Moreover, Nrf2 mature protein levels were significantly reduced in the H_2_O_2_ group as compared to the control group (*p* < 0.0001 vs. control group; Figure 2). Meanwhile, 2h pre-treatment with aloe extract significantly increased Nrf2 levels in the cells after exposure to H_2_O_2_ stimulus (*p* < 0.0001 vs. H_2_O_2_ group; Figure 2). In addition, it was revealed that catalase and SOD activity was decreased in HCE cells after exposure to H_2_O_2_ at a concentration of 500 μM for 24 h (*p* < 0.0001 versus control group) while 2h pre-treatment with aloe extract at 100 μg/mL significantly upregulated the catalase and SOD activity in HCE cells after exposure to H_2_O_2_ (*p* < 0.0001 vs. H_2_O_2_ group; Figure 2).

### 3.4. Effects of Aloe Extracts on Inflammatory Markers

**Figure 3:**
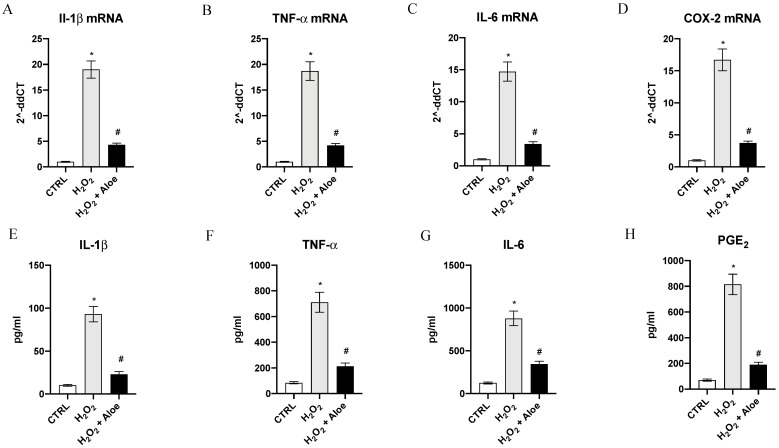
Effects of pre-treatment with aloe extract on IL-1β (**A**), TNF-α (**B**), IL-6 (**C**), COX-2 (**D**) mRNA expression. Effects of pre-treatment with aloe extract on IL-1β (**E**), TNF-α (**F**), IL-6 (**G**), PGE_2_ (**H**) levels. Values are expressed as the means ± SD. * *p* < 0.0001 vs. CTRL; # *p* < 0.0001 vs. H_2_O_2_.

As estimated, H_2_O_2_ induced a significant upregulation of mRNA expression of proinflammatory enzyme COX-2, a common feature of inflammation caused by oxidative stress, and a marked expression of proinflammatory cytokines IL-1β, IL-6 and TNF-α compared to control group (*p* < 0.0001 vs. control group; Figure 3). Pre-treatment with aloe extract in H_2_O_2_ stimulated HCE cells suppressed the increased mRNA for the inflammatory enzyme COX-2 and caused a marked reduction in the expression of the message of the inflammatory cytokine TNF-α, IL-6 and IL-1β (*p* < 0.0001 vs. H_2_O_2_ group; Figure 3). To confirm the anti-inflammatory effect of the aloe extract we measured the mature protein levels in the supernatants of HCE cells stimulated with H_2_O_2_. TNF-α, IL-6 and IL-1β levels were markedly increased (*p* < 0.0001 vs. control group; Figure 3). By contrast aloe extract pre-treatment blunted the increase of TNF-α, IL-6 and IL-1β in the HCE cells stimulated with H_2_O_2_ (*p* < 0.0001 vs. H_2_O_2_ group; Figure 3). PGE_2_ levels metabolite of COX-2 was also markedly released in the supernatants of HCE cells upon H_2_O_2_ stimulation (*p* < 0.0001 vs. control group; Figure 3). Pre-treatment with aloe extract resulted in a powerful reduction of PGE_2_ levels (*p* < 0.0001 vs. H_2_O_2_ group; Figure 3).

### 3.5. Effects of Aloe Extract on Apoptosis

**Figure 4:**
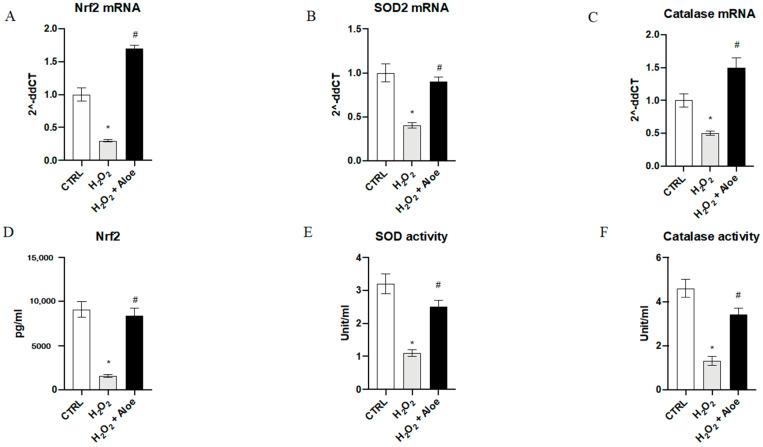
Effects of pre-treatment with aloe extract on Bcl-2 (**A**), Bax (**B**), Caspase-3 (**C**), Caspase-8 (**D**) mRNA expression. Effects of pre-treatment with aloe extract on Bcl-2 (**E**), Bax (**F**), Caspase-3 (**G**), Caspase-8 (**H**) levels. Values are expressed as the means ± SD. * *p* < 0.0001 vs. CTRL; # *p* < 0.0001 vs. H_2_O_2_.

To examine the role of aloe extract in regulating apoptosis in HCE cells stimulated with H_2_O_2_, the mRNA expression of Bcl-2, Bax, Caspase-3 and Caspase-8 was evaluated. HCE cells challenged with H_2_O_2_ for 24 h showed a marked reduction of Bcl-2 expression with a concomitant up-regulation in the mRNA expression of Bax, Caspase-3 and Caspase-8 compared to control cells (*p* < 0.0001 vs. control group; Figure 4). Conversely, when cells were pretreated with Aloe extract for 2 h, mRNA expression of Bcl-2, Bax, Caspase-3 and Caspase-8 were reversed (*p* < 0.0001 vs. H_2_O_2_ group; Figure 4). To better evaluate the antiapoptotic effect of aloe extract we measured the protein levels of Bcl-2, Bax, Caspase-3 and Caspase-8 in HCE cells challenged with H_2_O_2_. After HCE cells were challenged with H_2_O_2_ for 24 h Bcl-2 protein expression levels were significantly decreased with a contemporaneous increase in protein expression of Bax, Caspase-3 and Caspase-8 compared to control cells (*p* < 0.0001 vs. control group; Figure 4). By contrast, when cells were pretreated with aloe extract for 2 h, a significant growth in Bcl-2 protein expression with a simultaneous reduction of Bax, Caspase-3 and Caspase-8 protein levels were observed (*p* < 0.0001 vs. H_2_O_2_ group; Figure 4).

## 4. Discussion

Fuchs is a corneal endothelium degenerative condition characterized by the accumulation of focal guttae, contributing to oedema of the cornea and vision loss. The corneal endothelium is a single layer that serves as a barrier, maintains a particular level of corneal hydration and preserves corneal stromal clarity through precise spatial collagen fiber arrangement. End-stage FECD corneal endothelial cells are reduced in number and appear attenuated, inducing progressive stromal swelling and resulting in blurred vision. These pathological conditions are related to CECs apoptosis caused by an exaggerated ROS production that lead to oxidative stress. ROS can be divided into two forms: radical and non-radical species. Hydrogen peroxide (H_2_O_2_), superoxide anion (O2−), ozone (O_3_), and nitric oxide (NO) belong to the first category. Oxidative stress may have distinct effects, and the cellular response depends on its behaviour. The signalling role of ROS has also been shown to be important for the integrity of living organisms and their aging process. In addition, ROS may be involved in various damage mechanisms, such as membrane lipid peroxidation, protein structure damage and also in different ocular pathologies such as ocular surface disorders.

In this scenario, evaluating the efficacy of natural extracts could be of interest. Indeed, medicinal plants and their derivatives have shown several beneficial effects, including the reduction of reactive oxygen species (ROS) (antioxidant activity), the prevention of cell apoptosis, and the modulation of pro-inflammatory factors. In particular, among medicinal plants, *Aloe vera* (A. barbadensis Miller) has a number of pharmacological properties, such as antioxidant, immunomodulatory, bactericidal, antiviral, antifungal and anti-inflammatory due to the presence of a variety of chemicals like flavonoids, anthraquinones, enzymes, vitamins, and phenolic acids. In addition, in previous studies, *Aloe vera* extract showed the ability to speed up re-epithelialization and minimize fibrosis in superficial corneal lesions [24,41].

In light of these preceding observations, in the present study, we evaluated the efficacy of an extract from *Aloe vera* in an “in vitro” model of FECD, induced by H_2_O_2_ stimulus. Previous papers have investigated the possibility that hydrogen peroxide, its products, and/or other oxidant species may be in part responsible for the functional and structural alterations of corneal endothelial cells during ocular inflammatory disease processes [44]. Moreover, preceding works have demonstrated that the corneal endothelium is susceptible to oxidative stress, which leads to inflammation and apoptosis [5,45,46,47]. Our results have shown that hydrogen peroxide causes the alteration and apoptosis of corneal endothelial cells according to previous published papers [48,49]. Pre-incubation with aloe extract significantly reduced oxidative stress markers and upregulated the expression of Nrf2, SOD2 and Catalase when compared to cells cultures challenged with H_2_O_2_ alone. These effects may be attributed to Aloin, a substance with many biological activities present in *Aloe vera.* Indeed, Aloin has been demonstrated to have anti-oxidant effects in two different models of oxidative stress damage in skin fibroblasts and macrophages [27,50]. These results are in accordance with other studies that demonstrated the protective effects of Nrf2 agonists on oxidative stress in corneal endothelium due to the antioxidant activity of these compounds, corroborating the idea that the implementation of antioxidant systems may play a role in the treatment of corneal ROS related diseases [25].

Moreover, pre-treatment with aloe extract in H_2_O_2_ stimulated HCE cells, suppressed the increased mRNA for the COX-2 enzyme and caused a marked reduction in the expression of the message and protein levels of the inflammatory cytokine TNF-α, IL-6 and IL-1 β when compared to cell cultures challenged with H_2_O_2_ alone. These findings may be related to several Aloe compounds such as Aloin and Aloe-emodin that exhibited a great suppression of pro-inflammatory cytokine expression in murine macrophages and in human gingival fibroblast under oxidative stress stimulus [20,27]. Furthermore, our results agree with the findings of other previous studies showing that *Aloe vera*’s anti-inflammatory activity is linked to the downregulations of the arachidonic acid pathway via cyclooxygenase [51]. Moreover, it has been elucidated that the presence of sterols in the *Aloe vera* extract may reduce the production of the phospholipase A2 enzyme, which is accountable for the release of arachidonic acid precursors in the synthesis of prostaglandins. As a consequence, *Aloe vera* extract’s anti-inflammatory activity is the result of the inhibition of both prostaglandin and leukotriene synthesis [51]. Furthermore, it has been demonstrated that the herb’s extracts may inhibit the inflammatory process in different ways by reducing the number of circulating cytokines and also inhibiting the adhesion ability of leukocytes in the injury site. [52]. In addition, previous papers have demonstrated that medical herbal plants have antioxidant effects in human corneal cells and their administration to the eye influences local homeostasis among other effects on the cytokine network [53]. In particular, it has been well-demonstrated that *Aloe vera* extract is full of various biologically active constituents with different therapeutic properties, such as: Wound-healing properties and immunomodulatory, anti-inflammatory and antioxidant effects [20,54] Additionally, our results demonstrated that pre-treatment with *Aloe vera* extract for two hours increased Bcl-2 levels and reduced Bax, Caspase-3 and Caspase-8 mRNA expression and protein levels compared to cell cultures challenged with H_2_O_2_ alone. These results agree with the findings of previous studies showing that nature-derived antioxidant application had anti-inflammatory and anti-apoptotic activities, and may avoid the aggravation of different illnesses caused by oxidative stress in eye models [55,56,57].

Moreover, previous papers have already established that the activity of *Aloe vera* extracts did not have toxic effects even at high concentrations and can be helpful as a complimentary treatment for eye disorders. *Aloe vera* has well-established pharmacological proprieties that allow the activation and inhibition of different enzymes, modulating the metabolism of cells, the expression of anti-inflammatory, antifungal and antibacterial proprieties [58]. Additionally, in a previous study the healing of corneal epithelial lesions mechanically induced in rabbits after *Aloe vera* application was observed, without toxic effect [54,59]. Furthermore, *Aloe vera* extracts demonstrated a concentration-dependent ROS scavenging action that can be attributed to the phenolic compounds present in the extracts and this is considered its principal beneficial activity [60].

Nevertheless, the present study is subject to some limitations, for instance *Aloe vera*’s antioxidant properties are limited by simultaneous insufficient prevention of lipid peroxidation [61]. In addition, *Aloe vera* extracts have an inhibitory activity on NO production (which is also an effector radical in biological processes) because of constitutive and inducible NO synthase inhibition [62]. This effect could be a limitation because a proper level of NO allows the maintainence of homeostasis of the ocular surface and may work as a health preventive factor [63]. Furthermore, we evaluated only the effects of *Aloe vera* extract in this “in vitro” model of FECD, additional studies should be performed to compare the efficacy of this extract to other extracts from medical plants with antioxidant activities. Also, we did not asses in detail the potential ophthalmological side effects arising from human *Aloe vera* use, and such aspects should be carefully evaluated in future studies when considering a longer disease course and human lifespan.

To date there are no non-surgical treatments for FECD [64], but the present experiment demonstrated the efficacy of this extract from *Aloe vera* as treatment for FECD due to its modulating effect on the inflammatory cytokine network and antioxidant function, including ROS scavenging in eye tissue. Since, *Aloe vera* extracts are currently on the market for the treatment of other pathologies and severe side effects were not reported, the extract could be readily available for clinical trials in FECD patients. Therefore, these results exalt the potential future application of Aloe extract as a complimentary treatment option for patients with FECD, in a possible formulation of eye drops.

## 5. Conclusions

In conclusion, our findings suggest that the application of *Aloe vera* extract may protect the cornea from oxidative stress and may provide a scientific basis for the use of this extract in the treatment of corneal inflammation. This effect, in light of its high translational potential, merits confirmation in a clinical setting.

## Figures and Tables

**Table 1:** Primer list.

Gene	Gene Access Number	Sequence
β-actin	NG_007992	Fw:5′AGAGCTACGAGCTGCCTGAC3′
		Rw:5′AGCACTGTGTTGGCGTACAG3′
SOD2	NG_008729	Fw:5′GAGAAGTACCAGGAGGCGTTG3′
		Rw:5′GAGCCTTGGACACCAACAGAT3′
Catalase	NG_013339	Fw:5′ACTGAGGTCCACCCTGACTAC3′
		Rw:5′TCGCATTCTTAGGCTTCTCA3′
Nrf2	NC_000002.12	Fw:5′CTCCACAGAAGACCCCAACC3′
		Rw:5′TCTGCAATTCTGAGCAGCCA3′
IL-1β	NG_008851	Fw:5′TGAGCTCGCCAGTGAAATGA3′
		Rw:5′AGATTCGTAGCTGGATGCCG3′
TNF-α	NG_007462	Fw:5′CAGAGGGCCTGTACCTCATC3′
		Rw:5′GGAAGACCCCTCCCAGATAG3′
IL-6	NG_011640	Fw:5′TTCGGTCCAGTTGCCTTCTC3′
		Rw:5′CAGCTCTGGCTTGTTCCTCA3′
COX-2	NG_028206	Fw:5′GTTCCACCCGCAGTACAGAA3′
		Rw:5′AGGGCTTCAGCATAAAGCGT3′
Bcl-2	NG_009361	Fw:5′GCTCTTGAGATCTCCGGTTG3′
		Rw:5′AATGCATAAGGCAACGATCC3′
Bax	NG_012191	Fw:5′TTTGCTTCAGGGTTTCATCC3′
		Rw:5′CAGTTGAAGTTGCCGTCAGA3′
Caspase-3	NC_000004.12	Fw:5′CCTGGTTCATCCAGTCGCTT
		Rw:5′ TCTGTTGCCACCTTTCGGTT
Caspase-8	NC_007117.7	Fw:5′GGTTAGGGGACTCGGAGACT3′
		Rw:5′CAGGCTCAGGAACTTGAGGG3′

**Table 2:** Qualitative analyses of the phytochemical components of *Aloe vera* extract.

Reducing sugar
Phenolic compounds
Alkaloids
Flavonoids
Steroids and Terpenoids
Tannins
Glycosides
Anthraquinones

## Data Availability

The data presented in this study are available on request from the corresponding author.
